# Long‐term clinical outcomes and prognoses of ST‐segment elevation myocardial infarction patients who present with tombstoning ST‐segment elevation

**DOI:** 10.1111/anec.12725

**Published:** 2019-11-10

**Authors:** Veysel Ozan Tanık, Tufan Çınar, Barış Şimşek, Barış Güngör, İlker Avcı, İbrahim Halil Tanboga, Can Yücel Karabay

**Affiliations:** ^1^ Department of Cardiology Ankara Dışkapı Yıldırım Beyazıt Training and Research Hospital Ankara Turkey; ^2^ Department of Cardiology Sultan Abdülhamid Han Training and Research Hospital Health Science University Istanbul Turkey; ^3^ Department of Cardiology Dr. Siyami Ersek Thoracic and Cardiovascular Surgery Training and Research Hospital Health Science University Istanbul Turkey; ^4^ Department of Cardiology Hisar Hospital Intercontinental Istanbul Turkey

**Keywords:** long‐term clinical events, long‐term mortality, ST‐segment elevation myocardial infarction, Tombstoning ST‐segment elevation

## Abstract

**Introduction:**

Although patients with tombstoning ST‐segment elevation (Tomb‐ST) usually have poor in‐hospital and short‐term survival rates, no studies have examined the long‐term clinical outcomes and prognosis of ST‐segment elevation myocardial infarction (STEMI) patients who have this electrocardiographic pattern. Therefore, we aimed to evaluate the long‐term clinical events and mortality of such patients in this study.

**Methods:**

In this retrospective analysis, we included 335 consecutive patients who were diagnosed with acute anterior wall‐STEMI from January 2015 to June 2018. The criteria for the definition of Tomb‐ST were accepted as provided in a previous study. Endpoints of the study were the incidence of significant in‐hospital and long‐term major adverse clinical events (MACE) including the composite of total death, myocardial reinfarction, and hospitalizations due to heart failure.

**Results:**

Patients who presented with Tomb‐ST had significantly higher in‐hospital and long‐term mortality (10% [*n* = 12 patients] vs. 2.3% [*n* = 5 patients]; *p* < 0.001and 6.5% [*n* = 7 patients] vs. 1.9% [*n* = 4 patients]; *p* = .04, respectively). In a multivariate traditional and penalized Cox proportional hazard regression analysis, this type of electrocardiographic pattern was found as independent predictor of long‐term MACE (Odds ratio [OR]: 3.82, 95% confidence interval [CI]: 1.91–7.63, *p* < .001 and OR: 4.36, 95% CI: 1.97–9.66, *p* < .001, respectively).

**Conclusion:**

In the present study, we observed that the presence of Tomb‐ST might be an independent predictor of long‐term MACE in STEMI patients. To the best of our knowledge, this is the first study to evaluate the long‐term MACE of such patients.

## 1 INTRODUCTION

In current practice, electrocardiography (ECG) is the most commonly used test for the early diagnosis of ST‐segment elevation myocardial infarction (STEMI) (Garvey, Zegre‐Hemsey, Gregg, & Studnek, 2016). In addition to its important role in diagnosis, an ECG may provide additional information to further risk‐stratify patients (Nable & Brady, 2009). During the early phase of acute anterior wall‐STEMI, some variant changes in the amplitude and morphology of ST‐segment elevation may be observed in some patients. Tombstoning ST‐segment elevation (Tomb‐ST) in anterior precordial leads is one such morphologic variant that may indicate massive myocardial damage (Balci, 2009). In the current literature, a few observational studies have reported that patients with Tomb‐ST have a poor short‐term prognosis in addition to having a higher risk of in‐hospital adverse events (Ayhan et al., 2012; Balci & Yesildag, 2003; Kukla, Dudek, & Szczuka, 2006). Despite data regarding short‐term outcomes and prognosis, no study has examined the long‐term clinical outcomes and prognosis of STEMI patients with this electrocardiographic pattern. Therefore, we aimed to evaluate the long‐term clinical events and mortality of such patients in this study.

## 2 MATERIALS AND METHODS

### 2.1 Population

**Figure 1:**
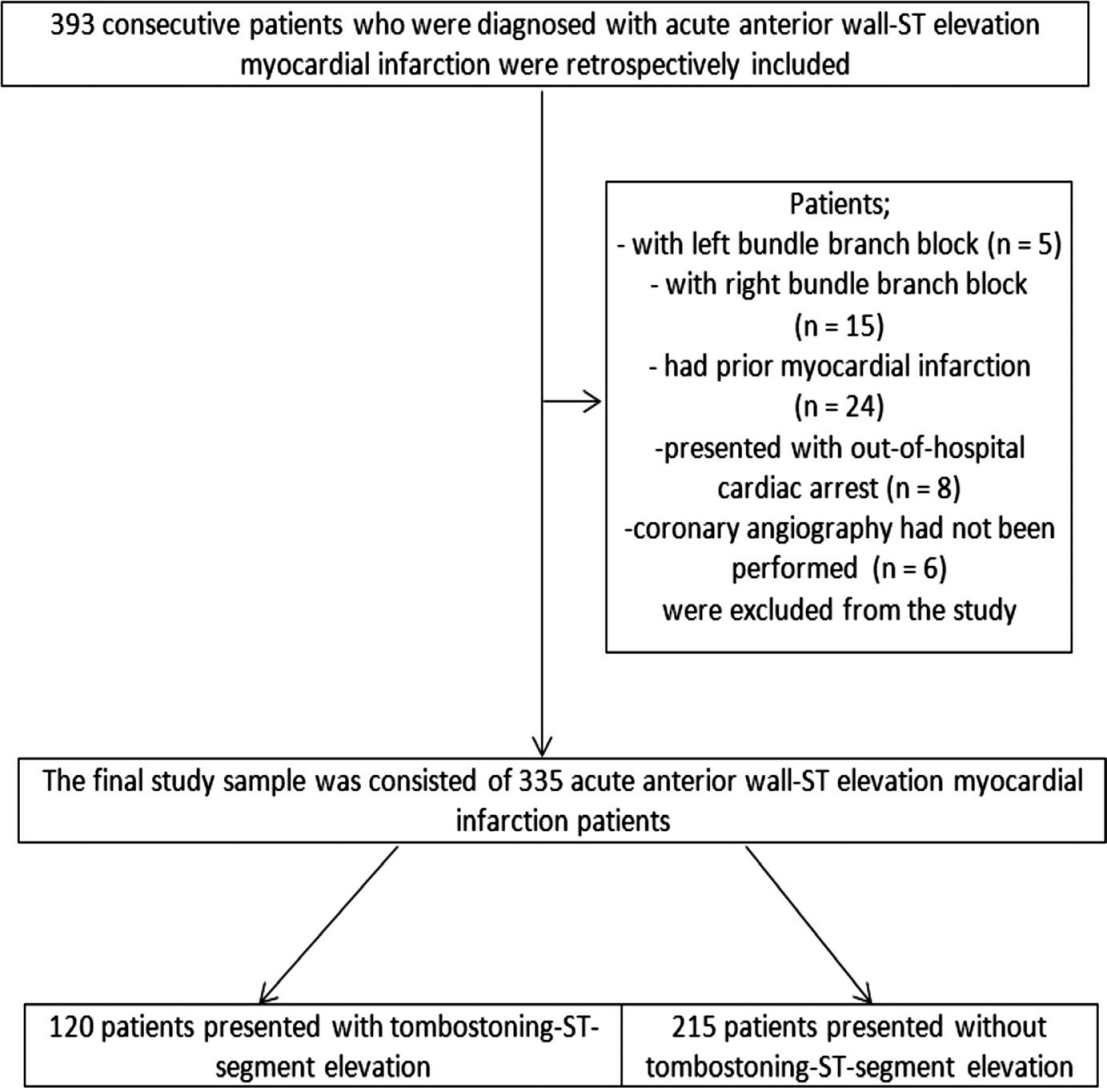
Flow chart of the study participants

We included 393 consecutive patients who were diagnosed with acute anterior wall‐STEMI from January 2015 to June 2018 in this retrospective analysis. Patients having either left bundle branch block (1.2% [*n* = 5 patients]) or right bundle branch block (3.8% [*n* = 15 patients]), a prior myocardial infarction (6.1% [*n* = 24 patients]), and who presented with out‐of‐hospital cardiac arrest (2.0% [*n* = 8 patients]) were excluded from the study (Figure 1). Notably, coronary angiography (CAG) had not been performed on 6 patients (1.5%); thus, these patients were not included in the study. The final study sample included 335 acute anterior wall‐STEMI patients. Clinical data, including age, gender, hypertension, diabetes mellitus, and current smoking status, were collected for each patient from medical records. The systolic and diastolic blood pressures and Killip class examination findings were accepted as the first data recorded on admission. In the present study, all patients were treated with the standard medical therapy, per current guidelines. The study was conducted in accordance with the “Good Clinical Practice” guidelines of the Declaration of Helsinki. Our study was reviewed and approved by the independent ethics committee (Approval number: HNEAH‐KAEK‐2018/106‐741).

### 2.2 Coronary angiography and percutaneous coronary intervention

Before performing CAG, a loading dose of antiplatelet medications, including 300 mg of aspirin and either 300–600 mg of clopidogrel or 180 mg of ticagrelor, were given to all patients. Primary percutaneous coronary intervention (PCI) was performed with standard techniques along with using the appropriate strategy by an experienced interventionist. The infusion of glycoprotein inhibitors IIb/IIIa and the choice of interventional equipment, including the balloon and the stent, were left to the operator's choice. An experienced cardiologist who was blinded to all clinical data analyzed the thrombolysis in myocardial infarction (TIMI) flow grade both before and after the intervention.

### 2.3 Laboratory analysis and echocardiographic examination

In all patients, blood samples were obtained immediately upon admission. An automatic analyzer was used to measure complete blood counts and biochemistry parameters. The total cholesterol, low‐density lipoprotein cholesterol, high‐density lipoprotein cholesterol, and triglycerides levels were obtained from the fasting blood samples the morning following admission. Serum levels of cardiac markers, including creatinine kinase myocardial band (CK‐MB), were measured at 12–24‐hr intervals until their peak values were obtained. All patients underwent a standard transthoracic echocardiographic examination within 24 hr of admission using a Vivid E5‐7 ultrasound system (GE Healthcare, Horten, Norway). Left ventricle ejection fraction (LVEF) was measured using the biplane Simpson method.

### 2.4 Endpoints

The endpoints of this study were the incidence of significant in‐hospital and long‐term major adverse clinical events (MACE). The following were considered as MACE during in‐hospital stay and follow‐up; the composite of total death, life threating arrhythmias including ventricular tachycardia (VT) and fibrillation (VF), hospitalization due to heart failure (HF), and myocardial reinfarction. In‐hospital mortality was defined as death from any cause during hospitalization. All long‐term MACE were obtained from medical records either during follow‐up visits or via telephone calls to either patients or their close relatives when follow‐up appointments could not be attended. The statewide death registry database was used to confirm patients who had died.

### 2.5 Definitions

**Figure 2:**
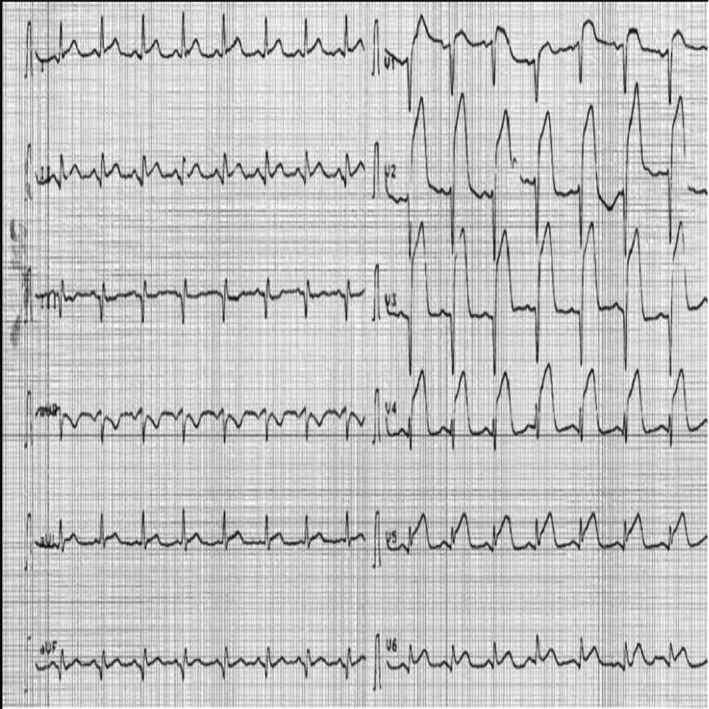
An example of patient who presented with tombostoning ST‐segment elevation

STEMI was determined by the universal definition of the myocardial infarction guideline of the European Society of Cardiology (Thygesen et al., 2018). The criteria for the definition of Tomb‐ST followed those proposed by Wimalaratna (1993) as follows: (a) The R wave is either absent or, if present, its duration is <0.04 s with a minimal amplitude, and there is no trough following the R wave; (b) the ST segment is convex upward and merges with either the descending limb of the R wave or the ascending limb of the QS/QR wave; (c) the peak of the convex ST segment is higher than whatever remains of the R wave; and (d) the convex ST segment merges with the ascending limb of the following T wave (Figure 2).

### 2.6 Statistical analysis

Continuous variables were presented as median and interquartile range, whereas categorical variables were presented as counts and percentages. The primary endpoint was MACE of the patients who were consecutively enrolled the study. The candidate predictors were tombstone sign, age, sex, etc. Kaplan–Meier analysis was performed to determine the cumulative events rates for patients with and without Tomb‐ST. The long‐rank test was performed to determine the cumulative event rates for patients with and without tombstone sign. Both traditional Cox proportional hazard regression and penalized maximum likelihood survival regression method was used to evaluate the relationship between outcome and candidate predictors. In our regression model, 7 candidate predictors were identified while outcomes were presented in 55 patients. Therefore, the penalized maximum likelihood estimation (PMLE) method was used to reduce the overfitting risk. A *p* value of <.05 indicated statistical significance. All statistical analyzes were performed using “RMS,” “Survival,” and “‘SurvMLP” packages with R‐Software v.3.5.1 (R Statistical Software, Institute for Statistics and Mathematics).

## 3 RESULTS

### 3.1 Patients’ characteristic, laboratory, and angiographic findings

In total, 335 acute anterior wall‐STEMI patients were included in this study (mean age was 58.1 ± 13.1, and 21.4% (*n* = 72) of the patients were female). We divided the study population into two groups: patients with and without Tomb‐ST. Their clinical baseline characteristics as well as laboratory and angiographic results are depicted in Table 1. The frequency of hypertension, diabetes mellitus, and smoking status were not significantly different between the groups (*p* > .05). On admission, the hemodynamic parameters of the systolic and diastolic blood pressures as well as total ischemia time were similar between the groups (*p* > .05). In terms of laboratory findings, the patients with Tomb‐ST had significantly higher admission cardiac troponin I and peak CK‐MB band levels (*p* < .05). Other laboratory findings were similar between the groups. A comparison of the angiographic findings revealed that the patients with Tomb‐ST had significantly higher rates of pre‐TIMI 0 flow before the procedure (*p* < .05).

**Table 1:** Baseline demographic characteristics, laboratory, and angiographic features of all patients with and without tombstoning ST‐segment elevation

	Tombstone (+) (*n*:120)	Tombstone (−) (*n*:215)	*p* value
Age, years	60.8 ± 13.4	57.5 ± 13.1	.03
Male, gender, *n* (%)	99 (82.5)	164 (76.2)	.21
History			
Hypertension, *n* (%)	54 (45)	79 (36.7)	.16
Diabetes mellitus, *n* (%)	31 (25.8)	46 (21.4)	.35
Current smoking status, *n* (%)	86 (71.6)	157 (73)	.89
At admission			
Systolic blood pressure, mm Hg	136 ± 29	138 ± 37	.49
Diastolic blood pressure, mm Hg	78 ± 17	81 ± 18	.14
Killip class 3–4, *n* (%)	7 (5.8)	6 (2.8)	.24
Total ischemic time, minute	140 (290)	150 (210)	.67
Admission laboratory variables			
Admission glucose, mg/dl	162 ± 73	148 ± 68	.11
Peak CK‐MB, ng/ml	85 (163)	36 (99)	<.001
Admission troponin I, ng/dl	19.9 (45.8)	2.3 (14.9)	<.001
Creatinine, mg/dl	0.91 ± 0.57	0.90 ± 0.41	.97
White blood cell count, cells/µl	13.8 ± 5.8	12.9 ± 4.8	.16
Hemoglobin, g/dl	13.8 ± 1.9	13.8 ± 2	.78
Triglyceride, mg/dl	135 ± 65	150 ± 85	.21
Total cholesterol, mg/dl	176 ± 42	181 ± 41	.32
LDL cholesterol, mg/dl	111 ± 35	116 ± 36	.31
HDL cholesterol, mg/dl	36 ± 8	35 ± 8	.86
Angiographic parameters			
Multivessel, *n* (%)	42 (35.0)	77 (35.8)	.91
Pre‐TIMI 0, *n* (%)	94 (78.3)	132 (61.4)	<.001
Post‐TIMI 3, *n* (%)	80 (66.6)	158 (73.4)	.21

Note

Continuous variables are presented as mean ± *SD*. Nominal variables presented as frequency (%).

Abbreviations: CK‐MB, creatinine kinase myocardial band; HDL, high‐density lipoprotein; LDL, low‐density lipoprotein; *n*, number; TIMI, thrombolysis in myocardial infarction.

### 3.2 In‐hospital MACE

Table 2 presents significant in‐hospital MACE of each group. The patients who presented with Tomb‐ST had a significantly higher incidence of VT and VF, inotrope use, and cardiopulmonary arrest. In patients with Tomb‐ST, the LVEF on hospital discharge was significantly lower. Notably, in‐hospital mortality was significantly elevated in patients with versus those without Tomb‐ST (10% [*n* = 12 patients] vs. 2.3% [*n* = 5 patients], respectively; *p* < .001).

**Table 2:** In‐hospital MACE of patients with and without tombstoning ST‐segment elevation

	Tombstone (+) (*n*:120)	Tombstone (−) (*n*:215)	*p* value
VT or VF, *n* (%)	21 (17.5)	16 (7.4)	<.001
CPA, *n* (%)	24 (20)	21 (9.7)	<.001
IV inotrope use, *n* (%)	31 (25.8)	29 (13.4)	<.001
LVEF on hospital discharge, %	39.6 ± 9.1	45.1 ± 10.5	<.001
Time of hospital stay, days	6.1 ± 4.6	6.3 ± 4.7	.71
In‐hospital mortality, *n* (%)	12 (10)	5 (2.3)	<.001

Note

Continuous variables are presented as mean ± *SD*. Nominal variables presented as frequency (%).

Abbreviations: CPA, cardiopulmonary arrest; LVEF, Left ventricular ejection fraction; MACE, major adverse clinical events; *n*, number; VF, ventricular fibrillation; VT, ventricular tachycardia.

### 3.3 Long‐term MACE

**Figure 3:**
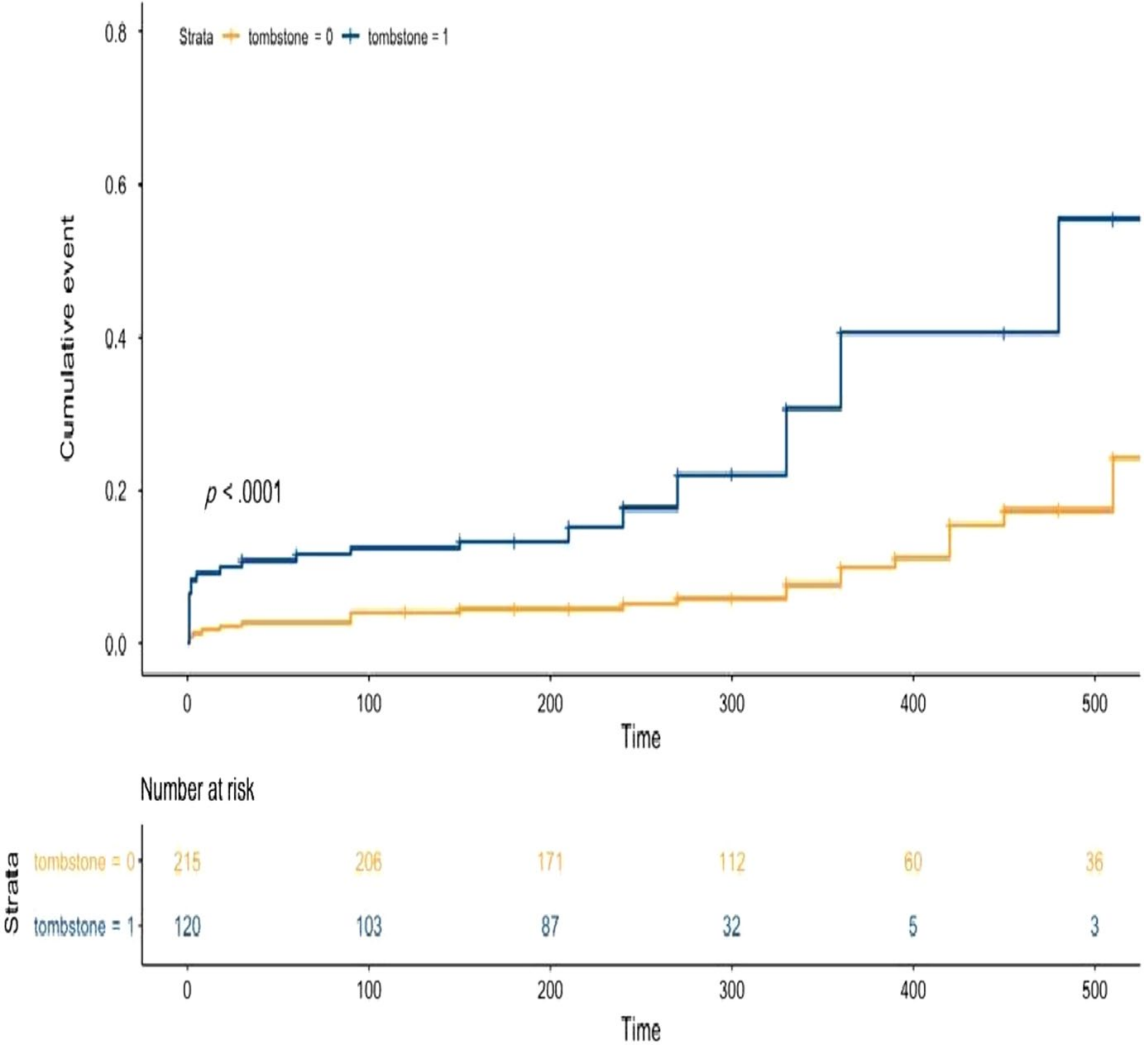
Cumulative event rates of patients with and without tombostoning ST‐segment elevation

The long‐term MACE of each group is shown in Table 3. The mean of follow‐up of the study was 15.2 ± 5.6 months. During long‐term follow‐up, both groups were similar in terms of hospitalization due to HF and myocardial reinfarction. We observed that patients with Tomb‐ST had poor survival rates compared to those without Tomb‐ST during long‐term follow‐up (6.5% [*n* = 7 patients] vs. 1.9% [*n* = 4 patients]; *p* = .04, respectively). Cumulative event rates of patients with Tomb‐ST were significantly greater than those without it [the long‐rank test = *p* < .001] (Figure 3).

**Table 3:** Long‐term MACE of patients with and without tombstoning ST‐segment elevation

	Tombstone (+) (*n*:108)	Tombstone (−) (*n*:210)	*p* value
Mortality, *n* (%)	7 (6.5)	4 (1.9)	.04
Myocardial reinfarction, *n* (%)	6 (5.5)	10 (4.7)	.79
HF requiring hospitalization, *n* (%)	6 (5.5)	8 (3.8)	.56
Follow‐up, months	14.9 ± 5.5	15.4 ± 5.8	.49

Note

Continuous variables are presented as mean ± *SD*. Nominal variables presented as frequency (%).

Abbreviations: HF, heart failure; MACE, major adverse clinical events; *n*, number.

### 3.4 Independent predictors of long‐term MACE

The effects of different variables on long‐term MACE were analyzed by using univariate, and then the traditional and penalized multivariate Cox proportional hazard regression analysis, as shown in Table 4. Age, male gender, a Killip class >2, Tomb‐ST, creatinine, systolic blood pressure, and post‐PCI TIMI flow <3 were found to be predictors of in‐hospital mortality by univariate analysis. In the traditional and penalized multivariate Cox proportional hazard regression analysis, male gender, post‐PCI TIMI flow <3, and Tomb‐ST (OR: 3.82, 95% CI: 1.91–7.63, *p* < .001 and OR: 4.36, 95% CI: 1.97–9.66, *p* < .001, respectively) were found to be independent predictors of long‐term MACE.

**Table 4:** Traditional and penalized Cox PH regression analysis for MACE

	Traditional Cox PH		Penalized Cox PH	
	*p* value	HR (95% CI)	*p* value	HR (95% CI)
Gender (male)	.015	0.41 (0.20–0.84)	.016	0.36 (0.15–0.82)
Age, years	.128	1.02 (0.99–1.04)	.185	1.02 (0.99–1.05)
Killip class	.065	1.48 (0.98–2.25)	.069	1.57 (0.96–2.54)
Post‐PCI TIMI flow	.001	0.63 (0.48–0.83)	<.001	0.55 (0.44–0.79)
Tomb‐ST	<.001	3.82 (1.91–7.63)	<.001	4.36 (1.97–9.66)
Creatinine	.661	1.11 (0.69–1.79)	.794	1.09 (0.55–2.15)
SBP	.254	0.99 (0.98–1.00)	.269	0.99 (0.97–1.01)

Abbreviations: CI, Confidence Interval; HR, Hazard Ratio; MACE, major adverse clinical events; PCI, percutaneous coronary intervention; PH, proportional hazard; SBP, Systolic blood pressure; TIMI, thrombolysis in myocardial infarction; Tomb‐ST, Tombstoning ST‐segment elevation.

## 4 DISCUSSION

The present study has showed that the Tomb‐ST may be an independent predictor of long‐term MACE in patients having this electrocardiographic pattern. We believed that this might be the first study to evaluate the long‐term MACE of patients who presented with Tomb‐ST.

Cardiovascular disease is still the leading cause of morbidity and mortality worldwide (Ibanez et al., 2018). Population‐based large observational studies have reported that the annual incidence of STEMI may range from 44–142/100,000 inhabitants in developed countries (Ibanez et al., 2018; Timmis et al., 2018). Even though there has been a significant decrease in the overall mortality of STEMI patients due to improvements in medical therapies and revascularization procedures, such as primary PCI, early risk assessment is paramount for these patients to receive appropriate and timely treatment.

Previous studies have demonstrated that ECGs might be used for risk stratification in patients who present with STEMI (Birnbaum & Sclarovsky, 2001; Nable & Brady, 2009). Several electrocardiographic parameters have been examined, including the magnitude of ST‐segment elevation, the T‐wave morphology, the presence of early repolarization, and fragmented QRS, on admission in patients with acute myocardial infarction (Atar & Birnbaum, 2005; Birnbaum et al., 2001; Güngör et al., 2016; Ozcan et al., 2014). Tomb‐ST is an electrocardiographic pattern of ST‐segment elevation in which the QRS complex, the ST segment, and the T wave merge to form a large upright monophasic deflection called a “tombstone” (Wimalaratna, 1993). Tomb‐ST is not an infrequent event; it is detected among 25%–30% of STEMI patients (Guo, Yap, Chen, Huang, & Camm, 2000; Tomcsányi et al., 2005). This electrocardiographic pattern is commonly seen in anterior localization (83.3%), even though it may sometimes be found in inferior localization (Guo et al., 2000). Although the underlying mechanisms that are responsible for a tombstone's appearance are unknown, some investigators have suggested that this type of ST‐segment elevation usually occurs due to the presence of multivessel disease with poor collateral circulation and lower LVEF (Balci, 2009; Guo et al., 2000).

Since the first description of Tomb‐ST by Wimalaratna (1993), few clinical studies have investigated the importance of this electrocardiographic pattern in STEMI patients. Tomcsányi et al. (2005) reported that the in‐hospital mortality rate of patients with Tomb‐ST in the PCI area was 13%, while our study found in‐hospital mortality to be 10%. Balci and Yesildag (2003) reported baseline characteristics and in‐hospital complications of patients both with and without Tomb‐ST; they concluded that the incidence of periinfarct angina symptoms and LVEF were significantly lower, and the incidence of death, cardiogenic shock, and life‐threatening arrhythmias, including VT and VF, occurred more frequently in patients with Tomb‐ST. Moreover, the peak CK‐MB levels, which can indicate infract size, have been found to be significantly elevated among these patients (Ayhan et al., 2012; Balci & Yesildag, 2003). Similar to the aforementioned study, we observed that patients with Tomb‐ST had higher peak levels of CK‐MB and cardiac troponin I in addition to having a higher incidence of adverse in‐hospital events than those without Tomb‐ST in our study.

Ayhan et al. (2012) recently examined in‐hospital and short‐term (6 months) adverse outcomes of patients with Tomb‐ST. They found Tomb‐ST to be an independent predictor of both in‐hospital and 6‐month all‐cause mortality. In addition, although myocardial reinfarction and TVR were observed to be similar between patients with and without Tomb‐ST during short‐term follow‐up, hospitalization due to HF was more frequent in patients with Tomb‐ST. In our study, in addition to the presence of a higher incidence of death in patients with Tomb‐ST during long‐term follow‐up, this type of electrocardiographic pattern was found to be an independent predictor of long‐term MACE.

Notably, our research differed somewhat from previously published studies. Our cohort was relatively larger, and the long‐term adverse clinical events of such patients were evaluated for the first time in the literature. We considered that as patients with Tomb‐ST had lower LVEF on hospital discharge, the presence of extensive myocardial damage with poor collateral circulation in an acute condition might explain increased long‐term MACE of Tomb‐ST patients. Based on the study findings, we believed that patients having this electrocardiographic finding may require close follow‐up in addition to having intensive medical treatment during long‐term follow‐up.

### 4.1 Study limitations

Our study had certain limitations, including its retrospective, cross‐sectional design. Although we attempted to enroll all consecutive STEMI patients, there is the possibility of selection bias. Because of the retrospective nature of the study, the underlying mechanisms of such events could not be well‐explained. Tomb‐ST usually occurs during the early stages of STEMI, meaning that some patients might not have this type of electrocardiographic pattern following admission to the emergency department. Although all patients were prescribed the standard medical therapy following discharge from hospital, there was the possibility of not standardization of this treatment during follow‐up.

## 5 CONCLUSION

In the present study, we demonstrated that patients with Tomb‐ST versus those without it might have higher in‐hospital fatal events and mortality, which was consistent with previous studies. However, this might be the first study to show that Tomb‐ST may be an independent predictor of long‐term MACE in STEMI patients.

## CONFLICT OF INTEREST

All authors declare that they do not have conflict of interest.
